# Editorial: Inflammatory Signaling in Bone Marrow Failure and Hematopoietic Malignancy

**DOI:** 10.3389/fimmu.2017.00660

**Published:** 2017-06-02

**Authors:** Eirini Trompouki, Lisa Mullen, Delmiro Fernandez-Reyes, Junji Yodoi, Soohyun Kim, Laura G. Schuettpelz

**Affiliations:** ^1^Max Planck Institute of Immunobiology and Epigenetics, Freiburg, Germany; ^2^Brighton and Sussex Medical School, Brighton, United Kingdom; ^3^University College London, London, United Kingdom; ^4^Kyoto University, Kyoto, Japan; ^5^Konkuk University, Seoul, South Korea; ^6^Washington University School of Medicine, St. Louis, MO, United States

**Keywords:** inflammation, bone marrow failure, hematopoietic malignancies, infection, leukemia

**The Editorial on the Research Topic**

**Inflammatory signaling in bone marrow failure and hematopoietic malignancy**

In response to inflammatory stimuli (e.g., infection and injury) hematopoietic stem and progenitor cells (HSPCs) proliferate and differentiate, giving rise to the effector cell types necessary to fight the acute insult. While this is an important part of the normal immune response, sustained or aberrant exposure to inflammatory signals can lead to a loss of HSPC function. Accumulating reports demonstrate that inflammatory cytokines (e.g., IFNγ, IFNα/β, and TNFα), and even pathogens or cell damage-associated ligands, can influence the function of HSPCs *via* both direct and indirect mechanisms and may contribute to clinically significant disorders of HSPCs including bone marrow (BM) failure and hematopoietic malignancies.

In this topic, we explore the effects of infections and their associated inflammatory cytokines on HSPCs. The seven reviews and one original article within this topic cover the role of inflammatory signals in regulating HSPCs from their ontogeny through old age and discuss the contribution of these signals to BM dysfunction and leukemogenesis. Furthermore, they highlight the unique effects of different pathogens and cytokines on HSPCs and discuss the direct, cell-autonomous effects of inflammation on HSPCs versus the indirect effects that are mediated by alterations in stromal cells and other cells within the BM microenvironment.

To start, a review by Clapes et al. discusses recent reports implicating a role for inflammatory signals in the emergence of hematopoietic stem cells (HSCs) during embryogenesis. Studies in zebrafish and mice have identified TNFα, IFNγ, IL1β, and Toll-like receptor 4 as important regulators of HSC ontogeny. These signals ultimately activate NF-κB, which is essential for HSC specification in the normal endothelial-to-hematopoietic transition. After their emergence, inflammatory signals continue to influence HSCs and progenitors throughout the lifetime of an organism, and, as discussed in a review by Kovtonyuk et al. on the “inflamm-aging” of hematopoiesis, they may contribute to the phenotype of aged HSPCs (e.g., decreased self-renewal and myeloid differentiation bias).

The next four articles in this topic focus on different aspects of how inflammatory signals affect HSPCs and contribute to BM suppression. Pascutti et al. discuss the direct and indirect impact of viral infections on HSPCs, noting that while such infections influence hematopoietic output in the short term, they may also contribute to BM pathologies such as cytopenias and malignancies (particularly when chronic). They point out that many different viral infections give rise to the same pathologies (e.g., pancytopenia), suggesting that common mechanisms of viral or immunological origin are responsible. On the other hand, they note that the same virus can have very different pathological manifestations in diverse individuals, suggesting that there are also genetic differences that contribute to the risk for BM pathologies in response to infection. Next, Smith et al. discuss the direct and indirect, or niche-mediated, effects of interferons on HSPCs, and their contribution to the pathogenesis of acquired aplastic anemia (AA). Patients with acquired AA have been shown to have elevated levels of IFNγ (1), which may impair HSPCs directly through a block in self-renewal and promotion of apoptosis, as well as indirectly *via* effects on cells of the microenvironment including macrophages, mesenchymal stromal cells, and T cells. The role of type I IFNs in AA is less clear; however, they may also both directly and indirectly affect HSPC function and may synergize with IFNγ in suppressing HSPCs and leading to BM failure. Highlighting the indirect effects of pathogens and inflammatory cytokines on HSPCs, a review by Nombela-Arrieta and Isringhausen focuses on the effects of these signals on the stromal cells of the BM microenvironment, noting that these effects are unique to individual pathogens and involve a variety of stromal cell subsets. For example, infections can target mesenchymal stromal cells, osteoblasts, endothelial cells, and others within the BM, influencing their survival and function *via* direct infection and/or indirect effects on supporting cytokines. Finally, Masouridi-Levrat et al. describe the contribution of chronic inflammation to poor graft function in individuals who have received an allogeneic HSC transplant. Inflammatory conditions such as graft-versus-host disease or infections can impair HSPC self-renewal and proliferation, in part, *via* upregulation of cytokines such as IFNγ and TNFα and the induction of apoptosis through Fas/Fas ligand signaling. Together, these four review articles emphasize the role of pathogens and inflammatory cytokines in BM suppression and suggest that interruption of these signals may have therapeutic benefit toward improving HSPC function in patients with chronic inflammatory conditions.

The last two papers in this topic focus on the contribution of inflammation to hematopoietic malignancy. Monlish et al. review the role of toll like receptor (TLR) signaling in myelodysplastic syndromes (MDS) and leukemias. Enhanced TLR expression and/or signaling has been described in multiple hematopoietic malignancies and may contribute to the pathogenesis of these diseases *via* both cell-autonomous and non-autonomous mechanisms. In the final article of the topic, Balandran et al. present original data demonstrating that mesenchymal stromal cells from pediatric patients with acute lymphoblastic leukemia differ from their normal counterparts, producing pro-inflammatory cytokines and displaying reduced amounts of CXCL12 and SCF. These changes, while detrimental to normal HSPCs, confer a growth advantage to leukemic precursor cells. Thus, these articles highlight aberrant inflammatory signaling in the premalignant cells and their microenvironment as potential therapeutic targets in patients with MDS and leukemia.

In summary, we have learned much in recent years regarding the effects of inflammatory signals on HSPCs and their contribution to BM failure and hematopoietic malignancies. However, as discussed throughout this topic, there are still open questions regarding the mechanisms by which these signals exert their effects, as well as understanding the balance between “normal” inflammatory signals that help shape the evolving immune system and contribute to an effective immune response to insult versus those that cause sustained damage to HSPCs. Continued research into these questions will hopefully lead to new therapeutic targets to improve BM function and inhibit transformation in patients with chronic or aberrant inflammation.

## Author Contributions

ET, LM, DF-R, JY, SK, and LS wrote and edited this manuscript.

## Conflict of Interest Statement

The authors declare that the research was conducted in the absence of any commercial or financial relationships that could be construed as a potential conflict of interest.
